# Quantitative assessment the longitudinal changes of pulmonary vascular counts in chronic obstructive pulmonary disease

**DOI:** 10.1186/s12931-022-01953-7

**Published:** 2022-02-14

**Authors:** Sang Won Park, Myoung-Nam Lim, Woo Jin Kim, So Hyeon Bak

**Affiliations:** 1 Department of Big Data Medical Convergence, School of Medicine, Kangwon National University, Chuncheon, Republic of Korea; 2 Department of Biomedical Research Institute, Kangwon National University Hospital, Chuncheon, Republic of Korea; 3 Department of Environmental Health Center, Kangwon National University Hospital, Chuncheon, Republic of Korea; 4 Department of Internal Medicine, School of Medicine, Kangwon National University, Chuncheon, Republic of Korea; 5 Department of Radiology, School of Medicine, Kangwon National University, 1 Kangwondaehak-gil, Chuncheon, Gangwon-do 24341 Republic of Korea

**Keywords:** Chronic obstructive pulmonary disease, Computed tomography, Longitudinal study, Pulmonary function tests, Pulmonary vascular

## Abstract

**Background:**

Chest computed tomography (CT) is a widely used method to assess morphological and dynamic abnormalities in chronic obstructive pulmonary disease (COPD). The small pulmonary vascular cross-section (CSA), quantitatively extracted from volumetric CT, is a reliable indicator for predicting pulmonary vascular changes. CSA is associated with the severity of symptoms, pulmonary function tests (PFT) and emphysema and in COPD patients the severity increases over time. We analyzed the correlation longitudinal changes in pulmonary vascular parameters with clinical parameters in COPD patients.

**Materials and methods:**

A total of 288 subjects with COPD were investigated during follow up period up to 6 years. CT images were classified into five subtypes from normal to severe emphysema according to percentage of low-attenuation areas less than -950 and -856 Hounsfield units (HU) on inspiratory and expiratory CT (LAA-950, LAA-856^exp^). Total number of vessels (N_total_) and total number of vessels with area less than 5 mm^2^ (N_<5 mm_) per 1 cm^2^ of lung surface area (LSA) were measured at 6 mm from the pleural surface.

**Results:**

N_total_/LSA and N_<5 mm_/LSA changed from 1.16 ± 0.27 to 0.87 ± 0.2 and from 1.02 ± 0.22 to 0.78 ± 0.22, respectively, during Global Initiative for Chronic Obstructive Lung Disease (GOLD) stage progression. Both parameters changed from normal to severe emphysema according to CT subtype from 1.39 ± 0.21 to 0.74 ± 0.17 and from 1.18 ± 0.19 to 0.67 ± 0.15, respectively. LAA-950 and LAA-856exp were negatively correlated with N_total_/LSA (r = − 0.738, − 0.529) and N_<5 mm_ /LSA (r = − 0.729, -− .497). On the other hand, pulmonary function test (PFT) results showed a weak correlation with N_total_/LSA and N_<5 mm_/LSA (r = 0.205, 0.210). The depth in CT subtypes for longitudinal change both N_total_/LSA and N_<5 mm_/LSA was (− 0.032, − 0.023) and (− 0.027) in normal and SAD, respectively.

**Conclusions:**

Quantitative computed tomography features faithfully reflected pulmonary vessel alterations, showing in particular that pulmonary vascular alteration started.

**Supplementary Information:**

The online version contains supplementary material available at 10.1186/s12931-022-01953-7.

## Background

Chronic obstructive pulmonary disease (COPD) is a multifaceted disease characterized by airflow obstruction, and is associated with chronic inflammatory response of the airways, often involving destruction of adjacent alveoli and vasculature [1, 2]. COPD have been known as a heterogeneous and complex condition with a variety of pathological and clinical compartments [3, 4]. Pulmonary vascular alteration is a major pathophysiological characteristic of COPD [5]. It is estimated that 30–70% of COPD patients have some degree of pulmonary vascular abnormalities due to pulmonary hypertension [6, 7]. Passive vascular compression by emphysema and hypoxic pulmonary vasoconstriction are thought to be critical for the pathogenesis of vascular changes, and recent studies have suggested that endothelial dysfunction is associated with vascular alterations in patients with COPD [2, 8, 9].

The gold standard for evaluating pulmonary vascular abnormality and hemodynamics is right heart catheterization, which is too invasive in clinical practice [2]. Angiographic studies of smokers showed narrowing and reduction of small pulmonary arteries in regions severely affected by emphysema [10, 11]. Chest computed tomography (CT) is widely used to evaluate the morphologic and dynamic abnormalities of COPD. The cross-sectional areas (CSAs) of the small pulmonary vessels, quantitatively extracted from volumetric CT, are reliable indicators of pulmonary vascular alteration [9]. CSAs are associated with symptoms, pulmonary function test (PFT), and severity of emphysema [5, 8, 9]. The extent of emphysema increases over time in patients with COPD [12]. However, there are few studies on the changes in vascular alterations during longitudinal follow-up in patients with COPD.

In this study, we conducted a quantitative analysis based on volumetric CT scans to identify vessel alterations in patients with COPD. The purpose of our study was to determine the differences in pulmonary vascular parameters measured by volumetric CT according to disease severity and CT phenotype, and to assess their correlations with clinical parameters. In addition, we observed longitudinal vascular changes in the subjects, classified by Global Initiative for Chronic Obstructive Lung Disease (GOLD) grade and CT subtype, during a follow-up period of up to 6 years.

## Methods

### Subjects

**Fig. 1:**
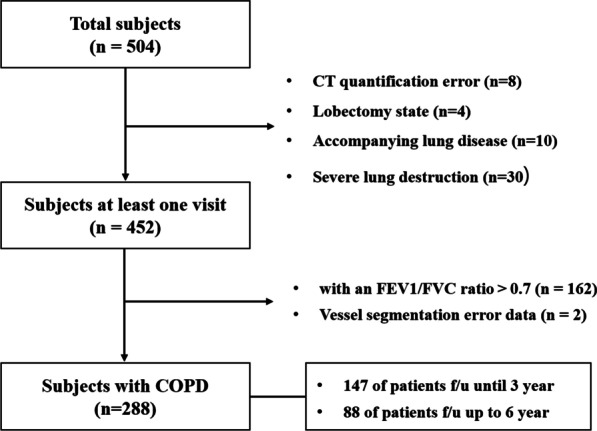
Selection of patients with chronic obstructive pulmonary disease (COPD). Patients who were visited at least once during the 6 year follow-up period were selected. *FEV1* forced expiratory volume in 1 s, *FVC* forced vital capacity

A total 504 of subjects were collected from the COPD in Dusty Areas (CODA) cohort, which consisted of Korean subjects residing near cement plants. As a prospective study, all subjects underwent medical interviews, PFTs, laboratory tests, and chest CT. COPD was diagnosed in subjects with post-bronchodilator forced expiratory volume in 1 s (FEV_1_) / forced vital capacity (FVC) ratio < 0.7 at baseline [13]. We excluded 206 subjects due to FEV_1_/FVC ratio ≥ 0.7 (n = 162), lung surgery (n = 4), CT quantification error (n = 10), and severe lung parenchymal distortion by tuberculosis sequelae and pneumoconiosis with progressive massive fibrosis (n = 30). Thus 288 subjects with COPD were finally investigated in the current study, of which 147 were investigated at least two chest CT scans within 3 years from baseline and 88 were followed up with CT scans at least two for up to 6 years (Fig. 1). Institutional Review Board approval for all processes of this study was obtained from Kangwon National University Hospital (KNUH 2012-06-007), and written informed consent was obtained from all subjects.

### Clinical and pulmonary function parameters

All subject data were obtained from interviews and assessments of physical condition using questionnaires, including demographic data, medical history, exposure environment, and respiratory symptoms. Dyspnea assessment was conducted using the modified Medical Research Council (mMRC) scale, and quality of life related to health was assessed by calculating the sum of scores on the subject-reported COPD Assessment Test (CAT).

PFTs were performed using the Easy One Kit (NDD, Zurich, Switzerland), before and after inhalation of 400 μg salbutamol. Specifically, the airflow limitation on spirometry for the severity of COPD is defined using the FEV1 and the FEV1/FVC ratio, and divided into four GOLD grades: grade 1 (≥ 80%), grade 2 (50–79%), grade 3 (30–49%), or grade 4 (< 30%)[13]. The number of subjects in grades 3 and 4 was insufficient compared to that of early stage patients, thus grades 3 and 4 were combined into one group.

### Chest CT acquisition

All volumetric CT scan images were obtained at full inspiration and expiration in the supine position. Intravenous contrast medium administration was not required. The CT scanners used in this study are first-generation dual-source CT scanners manufactured by Siemens Healthcare (Somatom Definition; Forchheim, Germany) with the following parameters:140 kVp, 100 mA, beam pitch 0.9–1, slice thickness 0.6 and 3 mm. All acquired CT images were reconstructed using the soft convolution kernel B30f.

### Quantitative analysis of CT images

**Fig. 2:**
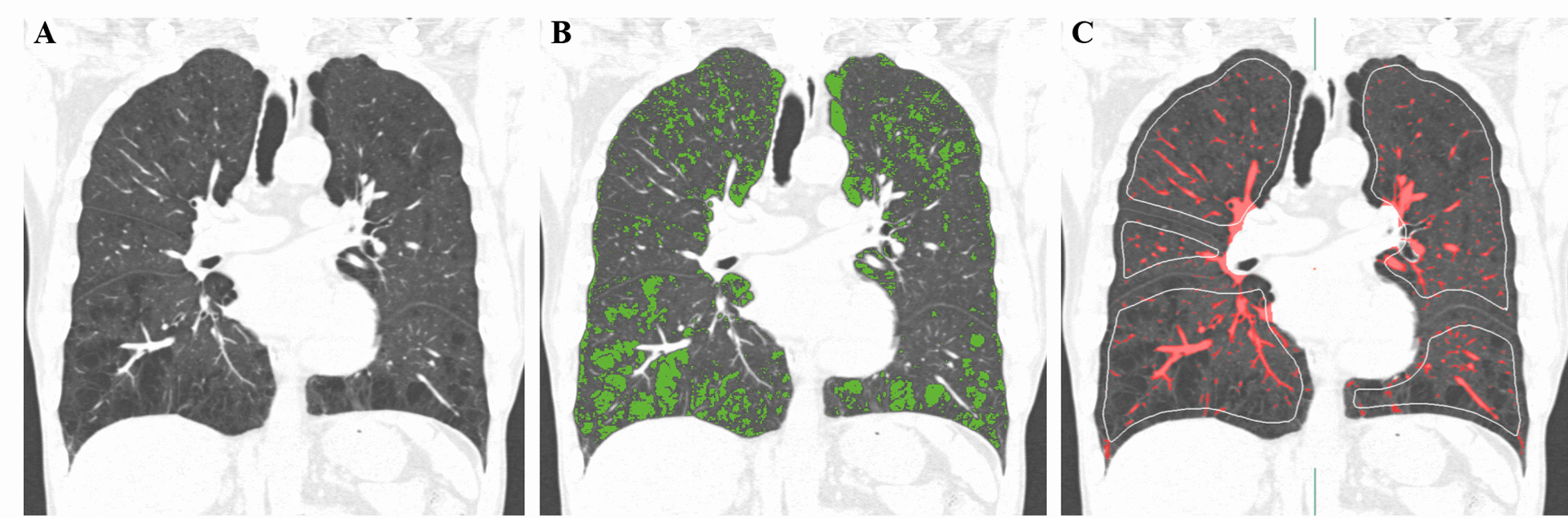
CT quantification of emphysema and pulmonary vessels. **A** CT coronal reconstructed image of a 54-year-old male with COPD GOLD grade 1 reveals centrilobular and paraseptal emphysema. **B** The emphysema was measured using a threshold of -950HU (shown in green; LAA-950, 12.3%). **C** Pulmonary vessels are automatically extracted and segmented (shown in red; Ntotal/LSA, 1.05; N5mm/LSA, 0.97), and the green contours show the lung surface area at 6 mm from the pleural surface. *CT* computed tomography, *LAA* low attenuation area

Lung segmentation and quantification of emphysema, air trapping, and pulmonary vessels were performed using an Aview® system (Coreline Soft Inc., Seoul, South Korea). The extent of emphysematous lung was measured by quantifying the fraction of low-attenuation areas less than -950 Hounsfield units (HU) on inspiratory CT scan (LAA-950) (Fig. 2). Air trapping was used to assess the percentage of low attenuation less than or equal to -856 HU measured on expiratory CT scan (LAA-856exp) [14]. CT images were classified into five subtypes according to LAA-950 and LAA-856exp: normal (LAA-950 < 5% and LAA-856exp < 20%), small airway disease (SAD, LAA-950 < 5% and LAA-856exp ≥ 20%), mild emphysema (LAA-950 ≥ 5% and < 10%), moderate emphysema (LAA-950 ≥ 10% and < 15%), and severe emphysema (LAA-950 ≥ 15%) [15].

The methodology for pulmonary vessel quantification is described in detail elsewhere [16]. Pulmonary vessels were extracted using a threshold of -750 HU. The extracted initial vessels were refined in detail as twigs of lung vascular structures using region growing and weighted minimum spanning tree (MST) algorithms with an orientation vector field. After pulmonary vascular structure reconstruction, the lung surface area (LSA) at a depth of 6 mm from the pleural surface was computed [17]. For each surface area, the total number of vessels (N_total_) and total number of vessels with vessel area less than 5 mm^2^ (N_< 5 mm_) were counted as robust values, and reported as values per 1 cm^2^ of LSA (N_total_/LSA; N_<5 mm_/LSA).

### Statistical analysis

Continuous variables were expressed as mean ± standard deviation. Differences between two groups were evaluated using independent sample t-tests and the chi-square statistics. One-way ANOVA was used to analyze the differences between the measured quantitative and qualitative pulmonary vessel changes and parameters. A mixed model was used to longitudinally analyze the changes in time of qualitatively and quantitatively measured pulmonary vessel parameters during the follow-up period of up to 6 years. The missing values that occurred in each patient during the entire follow-up period using the results within the remaining period were imputed to analyze the mixed model by multiple imputation (MI) method [18]. Pearson’s correlation analysis was used to measure the associations between pulmonary vessel parameters and clinical parameters such as FEV_1_, FEV_1_/FVC, CAT, LAA-950, and LAA-856exp. For all statistical analyses, p-values < 0.05 were considered statistically significant. All statistical analyses were performed using SAS (Version 9.4, SAS Institute, Cary, NC) and R (Version 3.6.3, The R Foundation for 211 Statistical Computing, Vienna, Austria; 64-bit platform).

## Results

### Patient characteristics

We present the demographic characteristics of the study cohort (Table 1 and Additional file 1: Table S1). In particular, by presenting the background of all subjects and subjects with smoking history, respectively, we considered the changes of vascular due to smoking. The 288 subjects included 236 (81.9%) men and 52 (18.1%) women. The mean age was 72.88 ± 7.27 (range 44.0 to 96.0) years. Regarding smoking history, the subjects included 71 non-smokers, 138 former smokers, and 76 current smokers, with mean pack-years 21.03 ± 25.49. The mean body mass index (BMI) of the subjects was 23.17 ± 3.13 kg/m^2^, the mMRC score 1.48 ± 1.16, and the CAT score 17.06 ± 9.68. The mean pulmonary function evaluation result (FEV_1_/FVC) was 58.80 ± 8.64.

**Table 1:** Characteristics of subjects with COPD in the CODA cohort

Characteristics	Baseline (n = 288)	3 year follow(n = 147)	6 year follow(n = 88)
Sex^1^			
Male	236 (81.9)	123(83.7)	74(84.1)
Female	52 (18.1)	24(16.3)	14(15.9)
Age, years^2^	72.88 ± 7.27	75.68 ± 6.53	78.00 ± 6.47
Smoking (n = 285)^1^			
Never	71 (24.9)	28(19.0)	17(19.5)
Former	138 (48.4)	92(62.6)	56(64.4)
Current	76 (26.7)	27(18.4)	14(16.1)
Pack-years^2^	21.03 ± 25.49		
BMI (kg/m^2^)^2^	23.17 ± 3.13	23.90 ± 3.50	23.32 ± 3.43
mMRC (n = 284)^2^	1.48 ± 1.16	1.24 ± 1.13	1.52 ± 1.06
CAT score (n = 284)^2^	17.06 ± 9.68	10.81 ± 6.75	13.32 ± 6.93
GOLD grade^1^			
1	147 (51.0)	61(47.7)	16(21.6)
2	118 (41.0)	58(45.3)	49(66.2)
3 and 4	23 (8.0)	9(7.0)	9(12.2)
PFE^2^			
FVC (L)	3.12 ± 0.80	3.04 ± 0.71	2.73 ± 0.66
FEV_1_ (L)	1.84 ± 0.56	1.81 ± 0.53	1.66 ± 0.50
FEV_1_/FVC(%)	58.80 ± 8.64	59.36 ± 9.81	60.75 ± 9.61

There were four and three non-responders among all patients for mMRC, CAT scores and Smoking, respectively

*BMI* Body Mass Index, *mMRC* modified Medical Research Council, *H.U* Hounsfield Unit, *GOLD* Global Initiative for Chronic Obstructive Lung Disease, *PFE* Pulmonary Function Evaluation, *CAT* Chronic obstructive pulmonary disease Assessment Test, *FEV1* Forced Expiratory Volume in 1 s, *FVC* Forced Vital Capacity

^1^Indicated data are number and percentages in parentheses

^2^Data are mean ± standard deviation (SD)

### Vessel quantification according to subtypes

Pulmonary vascular parameters were assessed according to the GOLD grade of the subjects (Table 2). The measured N_total_/LSA and N_<5 mm_/LSA decreased as the GOLD grade increased. N_total_/LSA was 1.16 ± 0.27 in GOLD 1, and decreased to 1.12 ± 0.29 and 0.87 ± 0.27 for GOLD 2 and 3/4, respectively. N_<5 mm_/LSA was 1.02 ± 0.22, 0.99 ± 0.23 and 0.78 ± 0.22 for GOLD 1, 2 and 3/4, respectively, thus more decreased than N_total_/LSA. The decrease of N_total_/LSA and that of N_<5 mm_/LSA were statistically significant (both p < 0.001).

**Table 2:** Vessel quantification based on GOLD severity criteria

	GOLD1 (n = 147)	GOLD2 (n = 118)	GOLD3,4 (n = 23)	p-value
N_total_/LSA	1.16 ± 0.27	1.12 ± 0.29	0.87 ± 0.27	< 0.001
N_<5 mm_/LSA	1.02 ± 0.22	0.99 ± 0.23	0.78 ± 0.22	< 0.001

*GOLD* Global Initiative for Chronic Obstructive Lung Disease, *N*_*total*_ Total number of vessels, *N*_*<5 mm*_ Total number of vessels with vessel area less than 5 mm^2^, *LSA* Lung surface area at 6 mm from the pleural surface

In addition, we measured pulmonary vascular parameters according to CT subtype (Table 3). The measured N_total_/LSA and N_<5 mm_/LSA were 1.39 ± 0.21 and 1.18 ± 0.19, respectively, in the normal CT subtype. Both N_total_/LSA and N_<5 mm_ /LSA decreased to 1.28 ± 0.17 and 1.12 ± 0.14, respectively, in the SAD subtype, and to 1.05 ± 0.19 and 0.95 ± 0.16 in the mild emphysema subtype. In the moderate and severe emphysema subtypes N_total_/LSA was 0.90 ± 0.18 and 0.74 ± 0.17, respectively, while N_<5 mm_/LSA was 0.82 ± 0.15 and 0.67 ± 0.15, showing more decreased numbers than N_total_/LSA for increasing emphysema severity. The decrease of both pulmonary vascular parameters was statistically significant (both p < 0.001).

**Table 3:** Vessel quantification based on CT subtypes

	Normal (n = 54)	SAD (n = 88)	Mild (n = 69)	Moderate (n = 34)	Severe (n = 43)	p-value
N_total_/LSA	1.39 ± 0.21	1.28 ± 0.17	1.05 ± 0.19	0.90 ± 0.18	0.74 ± 0.17	< 0.001
N_<5 mm_/LSA	1.18 ± 0.19	1.12 ± 0.14	0.95 ± 0.16	0.82 ± 0.15	0.67 ± 0.15	< 0.001

*N*_*total*_ Total number of vessels, *N*_*<5 mm*_ Total number of vessels with vessel area less than 5 mm^2^, *LSA* Lung surface area at 6 mm from the pleural surface, *SAD* Small airway disease

### Correlation between vessel parameters and clinical/quantitative CT parameters

We also investigated the correlation between the pulmonary vascular parameters and clinical/CT quantitative parameters (Table 4). FEV_1_ showed weak but significant positive correlation with N_total_/LSA and N_<5 mm_/LSA (correlation coefficient 0.205 and 0.210, respectively, both p < 0.001), and FEV_1_/FVC had a positive correlation with the same parameters (0.332 and 0.337 with N_total_/LSA and N_<5 mm_/LSA, respectively, both p < 0.001).

**Table 4:** Correlation between vessel parameters and clinical/quantitative CT parameters

	Post FEV_1_(%)	Post FEV_1_/FVC	Post FVC	CAT	LAA-950	LAA-856exp
N_total_/LSA	0.21^1^	0.33^1^	0.07	− 0.04	− 0.74^1^	− 0.53^1^
N_<5 mm_/LSA	0.21^1^	0.34^1^	0.08	− 0.08	− 0.73^1^	− 0.50^1^

*N*_*total*_ Total number of vessels, *N*_*<5 mm*_ Total number of vessels with vessel area less than 5 mm^2^, *LSA* Lung surface area at 6 mm from the pleural surface, *FEV*_*1*_ Forced expiratory volume in 1 s, *FVC* Forced vital capacity, *CAT* COPD Assessment Test, *LAA-950* Low-attenuation areas less than-950 Hounsfield unit on inspiration, *LAA-856* Low-attenuation areas less than-856 Hounsfield unit on expiration

^1^p < 0.001

LAA-950 and LAA-856exp showed strong negative correlation with N_total_/LSA and N_<5 mm_/LSA (LAA-950: correlation coefficients -0.738 and -0.729 with N_total_/LSA and N_<5 mm_/LSA, respectively; LAA-856^exp^: − 0.529 and − 0.497, p < 0.001). However, pulmonary vascular parameters had no statistically significant correlations with FVC and CAT scores.

### Longitudinal changes over a follow up period

**Fig. 3:**
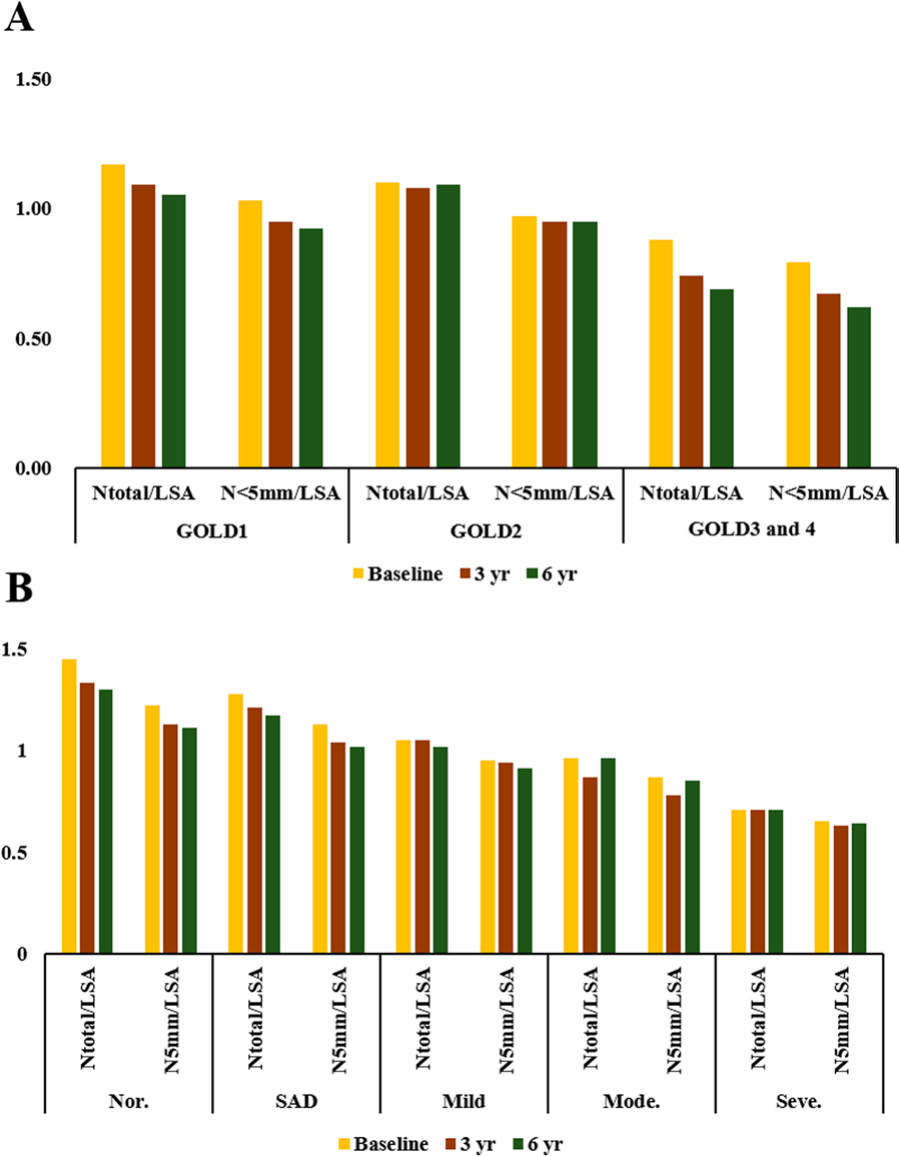
Longitudinal changes of vessel quantification during up to 6 years by GOLD grade and CT subtype. No significant differences were observed according to GOLD grade. However, the number of vessels during the initial COPD symptom progression (CT subtype normal to SAD) was markedly reduced, although without visually structural changes in the CT image. In other words, the decrease of vessels counts over 6 years was more pronounced in the airway disease phenotype than in the emphysema phenotype. *GOLD* Global Initiative for Chronic Obstructive Lung Disease, *CT* computed tomography, *SAD* small airway disease

We analyzed the pattern of pulmonary vascular parameter changes for the all subjects and subjects with smoking during the entire follow-up period of up to 6 years from baseline (Table 5 and Additional file 1: Table S2). Calibration was performed using covariates such as age, gender, and smoking status in individual subjects for the effective results. The results were presented as coefficients with 95% confidence interval (CI). Changes over time were observed according to CT subtypes and GOLD grades (Fig. 3). The longitudinal analysis of pulmonary vascular parameters showed a tendency for N_<5 mm_/LSA to decrease during the follow-up period as the severity increased from GOLD 1 to GOLD 3/4. However, the same pattern of change was not observed for N_total_/LSA, and neither vascular parameter showed a statistically significant change pattern.

**Table 5:** Longitudinal changes over a follow up period up to 6 years for all subjects

	GOLD severity				CT subtype					
	1	2	3,4	p	Normal	SAD	Mild	Moderate	Severe	p
N_total_/LSA	− 0.014 (− 0025, − 0.004)	− 0.007 (− 0.024, 0.009)	− 0.016 (− 0.038, 0.006)	0.61	− 0.032 (− 0.054, − 0.011)	− 0.023 (− 0.037, − 0.008)	− 0.005 (− 0.022, 0.013)	0.013 (− 0.021, 0.047)	− 0.003 (− 0.021, 0.015)	0.03
N_<5 mm_/LSA	− 0.019 (− 0.026, − 0.012)	− 0.014 (− 0.020, − 0.007)	− 0.006 (− 0.019, 0.008)	0.30	− 0.027 (− 0.041, − 0.013)	− 0.027 (− 0.034, − 0.019)	− 0.008 (− 0.014, − 0.001)	0.001 (− 0.015, 0.017)	− 0.005 (− 0.016, 0.005)	0.40

*GOLD* Global Initiative for Chronic Obstructive Lung Disease; *N*_*total*_ Total number of vessels, *N*_*<5 mm*_ Total number of vessels with vessel area less than 5 mm^2^; *LSA* Lung surface area at 6 mm from the pleural surface, *SAD* Small airway disease

Unlike GOLD grades, CT subtypes based on volumetric quantitative analysis results of emphysema and air trapping severity would clearly indicate a decline. The CT image-based quantitative volumetric scan results reflect the longitudinal changes over a follow-up period of up to 6 years of the N_total_/LSA and N_<5 mm_/LSA from the normal to the severe stage (Additional file 1: Tables S3 and S4). Both pulmonary vascular parameters exhibited a more pronounced decrease from normal to mild, than from moderate to severe. The results of depth of longitudinal change in Ntotal /LSA progressed − 0.032 and -0.023 in normal and SAD, respectively. In addition, the change in N<5mm/LSA was − 0.027 and − 0.027 in the same subtypes. Moreover, only N_total_/LSA showed a statistically significant result (p value of 0.031) over the entire follow-up period.

## Discussion

In this study, we performed a quantitative analysis of pulmonary vessel changes according to GOLD grade and CT subtype. As the GOLD grade based on PFT and the emphysema severity based on volumetric chest CT increased, the number of pulmonary vessels consistently decreased. In addition, quantitative longitudinal analysis up to 6 years demonstrated that the number of vessels decreased more significantly in the normal and SAD subtypes than in the emphysema subtypes, while no significant differences were observed according to GOLD grades.

Pulmonary vascular alteration is an important complication in the natural history of COPD, but its pathophysiologic mechanisms are still poorly understood [19]. Endothelial dysfunction is a major cause of vascular remodeling and emphysema [2, 8, 20]. Chest CT could quantitatively assess macroscopic pulmonary vascular alterations in subjects with COPD. The ratio of the main pulmonary artery to the ascending aorta diameter has been suggested as an important marker for pulmonary vascular disease [19]. Approximately 66% of subjects with COPD have some degree of pulmonary hypertension, and a pulmonary artery-to-ascending-aorta ratio > 1 was associated with acute exacerbation (AE) and mortality in patients with COPD [21–23]. Our previous study showed that the pulmonary artery-to-ascending aorta ratio was correlated with FEV_1_ in patients with mild to moderate COPD [24]. In addition, a study using CT and cardiac magnetic resonance imaging reported that pulmonary artery enlargement is associated with the loss of blood volume in the distal pulmonary vessels in patients with COPD [25].

The CSAs of the small pulmonary vessels can be evaluated quantitatively on CT to identify pulmonary vascular alterations in patients with COPD [19, 26]. Several studies found the CSAs of small pulmonary vessels to be associated with symptoms, pulmonary artery pressure, pulmonary function, exercise capacity, AE of COPD, and mortality [5, 27, 28]. In our study, similar to other studies, N_total_/LSA and N_<5 mm_/LSA showed a distinct decrease as the GOLD grades progressed. Histological studies have shown that a greater degree of emphysema and SAD are associated with pulmonary vascular alteration [29, 30]. Downregulation of lung vascular endothelial growth factor (VEGF) and upregulation of inducible nitric oxide synthase (iNOS), which can lead to endothelial dysfunction, play crucial roles in the development of vascular alteration and emphysema [6, 20, 31]. Previous studies have found a relationship between quantitative CT vascular parameters and emphysema [8, 17]. Likewise, the current study showed that N_total_/LSA and N_<5 mm_/LSA were negatively correlated with LAA-950. In addition, the quantitative assessment of pulmonary vascular alterations may be more strongly associated with the extent of emphysema than the PFT results.

COPD is a heterogeneous disease with various clinical and pathologic characteristics, and can traditionally be distinguished into two phenotypes: emphysema and airway disease [32, 33]. VEGF is a potential mediator of pulmonary vascular remodeling, and its expression increases in the airway of bronchitis-type patients, leading to abnormal proliferation of endothelial and vascular smooth muscle cells in pulmonary vessels [34]. A study reported that pulmonary vascular alteration was more strongly associated to the emphysema phenotype than to the bronchitis phenotype in patients with COPD [8]. In our study, the analysis was conducted by dividing the patients into five subtypes based on quantitative CT analysis. Compared with the emphysema phenotype, N_total_/LSA and N_<5 mm_/LSA were significantly higher in the SAD phenotype.

In the past, pulmonary vascular disease was considered an end-stage feature of COPD, and pulmonary hypertension was observed in 90% of patients with GOLD grade 4 COPD [17, 35]. However, recent studies have shown that pulmonary vascular alteration occurs in the setting of subclinical and early stage COPD by an impairment of endothelial function in pulmonary vessels [4, 19, 26, 36, 37]. Emphysema and air trapping progressed over time in smokers [12]. Some studies reported that emphysema increased over 2–3 years, whereas the CSAs of small pulmonary vessels did not decrease [9, 38]. In our study, there were no changes in each GOLD grade, but the number of vessels decreased in the normal and SAD CT subtype over the follow-up period of up to 6 years. This suggests that vessel changes over time were more prominent in the normal and SAD phenotype than in the emphysema phenotype. However, various factors could have affected this result, because the pulmonary hemodynamics affecting quantitative CSA assessment can be changed by breath-holding, circulating blood volume, and treatment [9, 38, 39].

This study has several limitations. First, we quantitatively measured the pulmonary vessel count change based on volumetric chest CT but could not distinguish between the pulmonary artery and vein. Second, the gold standard for assessing pulmonary vascular abnormality and pulmonary hypertension is right heart catheterization, but this was not done in our study because of the invasiveness of the method. Third, a longitudinal analysis was performed over the 6-year follow-up period, but the number of subjects gradually decreased. Thus, we performed to analyze after missing value correction using the MI method [18]. However, the statistical power to detect statistical significance in longitudinal observations of pulmonary vascular changes was lack. In addition, we considered quantitatively and qualitatively the emphysema index for all subjects and subjects with smoking to observe longitudinal change but the results were not shown the significance of statistical results. Therefore, validation of our results in a large cohort study is necessary. The last one is that the cohort used in this study was collected for subjects in a dust area. In order words, it includes non-smoking COPD caused by dust other than cigarette smoking, generalization might be limited. In addition, 52 subjects with asthma were included but the diagnosis of asthma is unclear due to collecting based on questionnaire survey. Therefore, it was difficult to exclude asthma patients.

## Conclusion

Quantitative pulmonary vascular parameters measured using volumetric chest CT were significantly associated with clinical measures of COPD severity. Quantitative CT features faithfully reflected pulmonary vessel alterations in patients with COPD. In addition, we performed a longitudinal analysis of pulmonary vessel changes for up to 6 years according to GOLD grade and CT subtype. The long-term follow-up revealed that pulmonary vessel change was more severe in the normal and SAD subtype than in the emphysema subtype.

## Supplementary Information

**Additional file 1: Table S1.** Characteristics of subjects with COPD in the CODA cohort (Smoking group). **Table S2.** Longitudinal changes over a follow up period up to 6 years for subjects with smoking. **Table S3.** The GOLD results reflecting the longitudinal changes of pulmonary vascular up to 6 years. **Table S4.** The CT-based results reflecting the longitudinal changes of pulmonary vascular up to 6 years

## Data Availability

The datasets used for the current study are available from the corresponding author on reasonable request.

## Abbreviations

AE: Acute exacerbation
CAT: COPD assessment test
CODA: COPD in dusty area
COPD: Chronic obstructive lung disease
CSAs: Cross-sectional areas
CT: Computed tomography
EI: CT-emphysema index
FEV_1_: Forced expiratory volume in 1 s
FVC: Forced vital capacity
GOLD: Global initiative for obstructive lung disease
HU: Hounsfield unit
iNOS: Inducible nitric oxide synthase (iNOS)
LAA-950: Low-Attenuation Areas less than-950
LSA: Lung surface area
mMRC: Modified medical research council
N_total_: Total number of vessels as values per 1 cm^2^
N_<__5__ mm_: Total number of vessels with vessel area less than 5 mm^2^
PFT: Pulmonary function test
VEGF: Lung vascular endothelial growth factor
