# Supplementary material for: Quantitative assessment the longitudinal changes of pulmonary vascular counts in chronic obstructive pulmonary disease
Source: Respir Res. 2022 Feb 14;23:29. doi: 10.1186/s12931-022-01953-7 (PMC8842934; doi:10.1186/s12931-022-01953-7)
Supplement: Supplementary file 1 — Additional file 1: Table S1. Characteristics of subjects with COPD in the CODA cohort (Smoking group). Table S2. Longitudinal changes over a follow up period up to 6 years for subjects with smoking. Table S3. The GOLD results reflecting the longitudinal changes of pulmonary vascular up to 6 years. Table S4. The CT-based results reflecting the longitudinal changes of pulmonary vascular up to 6 years [file 12931_2022_1953_MOESM1_ESM.docx]

Additional file 1: Table S1. Characteristics of subjects with COPD in the CODA cohort (Smoking group).

| **Characteristics** | **Baseline (n = 214)** | **3 year follow(n=119)** | **6 year follow(n=70)** |
| --- | --- | --- | --- |
| **Sex^1^** |  |  |  |
| Male | 211(98.6) | 117(98.3) | 68(97.1) |
| Female | 3(1.4) | 2(1.7) | 2(2.9) |
| **Age, years^2^** | 72.59 ± 7.12 | 75.26 ± 6.91 | 77.76 ± 6.60 |
| **Smoking ^1^** |  |  |  |
| Former | 138 (48.4) | 92(77.3) | 56(80.0) |
| Current | 76 (26.7) | 27(22.7) | 14(20.0) |
| **Pack-years(n=210)^2^** | 28.14 ± 25.88 |  |  |
| **BMI (kg/m^2^)^2^** | 22.75 ± 3.11 | 23.69 ± 3.59 | 23.09 ± 3.53 |
| **mMRC (n=210)^2^** | 1.40 ± 1.12 | 1.13 ± 1.10 | 1.47 ± 1.00 |
| **CAT score (n=210)^2^** | 16.53 ± 9.54 | 10.22 ± 6.89 | 13.13 ± 6.40 |
| **GOLD grade^1^** |  |  |  |
| 1 | 106(49.5) | 45(43.7) | 12(20.3) |
| 2 | 89(41.6) | 50(48.5) | 39(66.1) |
| 3 and 4 | 19(8.9) | 8(7.8) | 8(13.6) |
| **PFE^2^** |  |  |  |
| FVC (L) | 3.29 ± 0.72 | 3.21 ± 0.63 | 2.87 ± 0.57 |
| FEV_1_ (L) | 1.94 ± 0.55 | 1.90 ± 0.51 | 1.75 ± 0.48 |
| FEV_1_/FVC(%) | 58.38 ± 9.06 | 59.02 ± 10.19 | 60.83 ± 9.90 |

^1^Indicated data are number and percentages in parentheses.
^2^ Data are mean ± standard deviation (SD).

There were four and three non-responders among all patients for mMRC, CAT scores and Smoking, respectively.

Abbreviations: BMI; Body Mass Index, mMRC; modified Medical Research Council, H.U; Hounsfield Unit, GOLD; Global Initiative for Chronic Obstructive Lung Disease, PFE; Pulmonary Function Evaluation, CAT; Chronic obstructive pulmonary disease Assessment Test, FEV1; Forced Expiratory Volume in 1 s, FVC; Forced Vital Capacity,

Additional file 1: Table S2. **Longitudinal changes over a follow up period up to 6 years for subjects with smoking.**

|  | **GOLD severity** | | | | **CT subtype** | | | | | |
| --- | --- | --- | --- | --- | --- | --- | --- | --- | --- | --- |
|  | 1 | 2 | 3,4 | p | Normal | SAD | Mild | Moderate | Severe | p |
| N_total_/LSA | -0.008  (-0.019, 0.002) | -0.005  (-0.018, -0.007) | -0.007  (-0.037, 0.023) | 0.43 | -0.017  (-0.037, 0.004) | -0.014  (-0.034, 0.007) | 0.001  (-0.018, 0.020) | 0.020  (-0.112, 0.152) | -0.001  (-0.021, 0.018) | 0.02 |
| N_<5mm_/LSA | -0.009  (-0019, -0.0001) | -0.006  (-0.015, 0.004) | -0.008  (-0.036, 0.019) | 0.52 | -0.015  (-0.033, 0.003) | -0.015  (-0.033, 0.004) | -0.003  (-0.019, 0.013) | 0.012  (-0.092, 0.116) | -0.004  (-0.022, 0.014) | 0.07 |

Abbreviations: GOLD; Global Initiative for Chronic Obstructive Lung Disease; N_total_; Total number of vessels; N_<5 mm_; Total number of vessels with vessel area less than 5 mm^2^; LSA; Lung surface area at 6 mm from the pleural surface; SAD; Small airway disease.

Additional file 1: Table S3. The GOLD results reflecting the longitudinal changes of pulmonary vascular up to 6 years.

|  | **GOLD1(n=147)** | | **GOLD2(n=118)** | | **GOLD3(n=23)** | | **p-value** | |
| --- | --- | --- | --- | --- | --- | --- | --- | --- |
| **3 yr f/u** | | | | | | | | |
| **N_total_/LSA** | | 1.10±0.27 | | 1.11±0.26 | | 0.82±0.26 | | 0.0023 |
| **N_<5mm_/LSA** | | 0.96±0.22 | | 0.97±0.20 | | 0.72±0.21 | | 0.0010 |
| **6 yr f/u** | | | | | | | | |
| **N_total_/LSA** | | 1.06±0.17 | | 1.08±0.22 | | 0.70±0.18 | | 0.0023 |
| **N_<5mm_/LSA** | | 0.92±0.14 | | 0.95±0.19 | | 0.62±0.17 | | 0.0010 |

Abbreviations: GOLD; Global Initiative for Chronic Obstructive Lung Disease, N_total_; Total number of vessels, N_<5 mm_; Total number of vessels with vessel area less than 5 mm^2^, LSA; Lung surface area at 6 mm from the pleural surface.

Additional file 1: Table S4. The CT-based results reflecting the longitudinal changes of pulmonary vascular up to 6 years.

|  | **no(n=24)** | **SAD(n=46)** | **mild(n=44)** | **moderate(n=14)** | **severe(n=19)** | **p-value** |
| --- | --- | --- | --- | --- | --- | --- |
| **3 yr f/u** | | | | | | |
| **Nt_total_/LSA** | 1.27±0.31 | 1.20±0.21 | 1.06±0.18 | 0.89±0.15 | 0.73±0.20 | <.0001 |
| **N_<5mm_ /LSA** | 1.08±0.26 | 1.04±0.16 | 0.94±0.15 | 0.80±012 | 0.65±0.18 | <.0001 |
| **6 yr f/u** | | | | | | |
| **N_total_/LSA** | 1.15±0.17 | 1.11±0.17 | 1.03±018 | 1.02±0.21 | 0.72±0.23 | <.0001 |
| **N_<5mm_ /LSA** | 0.99±0.15 | 0.97±0.15 | 0.92±0.14 | 0.89±0.18 | 0.63±0.19 | <.0001 |

Abbreviations: N_total_; Total number of vessels, N_<5 mm_; Total number of vessels with vessel area less than 5 mm^2^, LSA; Lung surface area at 6 mm from the pleural surface; SAD; Small airway disease. LSA.
